# Prevalence of *Aspergillus* Section *Nigri* Complex Species Isolated from Clinical Specimens in Kuwait and Their Susceptibility to Antifungal Drugs

**DOI:** 10.3390/jof12060430

**Published:** 2026-06-12

**Authors:** Mohammad Asadzadeh, Ziauddin Khan, Suhail Ahmad

**Affiliations:** Department of Microbiology, Faculty of Medicine, Kuwait University, Safat 13110, Kuwait; mohammad.assadzadeh@ku.edu.kw (M.A.); ziauddin381044@gmail.com (Z.K.)

**Keywords:** *Aspergillus* section *Nigri*, *A. niger*, *A. tubingensis*, prevalence in Kuwait, clinical samples

## Abstract

*Aspergillus* spp. are common environmental molds worldwide and mostly cause infections in immunocompromised individuals. We have previously shown that black aspergilli (*Aspergillus* section *Nigri*) isolates are the most common aspergilli in indoor and outdoor hospital environments in Kuwait. This study reports on the relative prevalence of different *Aspergillus* section *Nigri* complex species among clinical isolates and their susceptibility to antifungal drugs. Black aspergilli isolated from clinical (n = 34) and environmental (n = 2) sources were studied. The isolates were initially identified as *Aspergillus* section *Nigri* complex members based on morphological characteristics. Species-specific identification was carried out by PCR-sequencing of the β-tubulin gene fragment and sequence comparisons with the GenBank database. The phylogenetic analysis was performed by using the Maximum Likelihood method in MEGA (version 11) software. Antifungal susceptibility testing was performed by Etest. The phylogenetic analysis based on β-tubulin gene sequences identified only three species: *A. niger sensu stricto* (*A. niger*) (n = 26), *A. tubingensis* (n = 7), and *A. luchuensis* (n = 1) among 34 clinical *Aspergillus* section *Nigri* isolates in Kuwait. All seven otomycoses cases were due to *A. niger*. The two environmental isolates were identified as *A. niger* and *A. tubingensis*. All isolates appeared susceptible to all five (amphotericin B, itraconazole, voriconazole, posaconazole, and caspofungin) antifungal drugs tested. In conclusion, our study shows that *A. niger* predominates among phenotypically identified clinical isolates of the *Aspergillus* section *Nigri* complex, and that *A. niger* is also the main agent in otomycosis cases in Kuwait. The detection of only three species within the *Aspergillus* section *Nigri* complex in Kuwait could be due to the limited number (n = 34) of clinical isolates analyzed in this study.

## 1. Introduction

*Aspergillus* species are among the common environmental molds worldwide and mostly cause infections in immunocompromised individuals [1,2,3]. Human aspergillosis includes a broad range of disease presentation varying in severity from life threatening invasive pulmonary aspergillosis (IPA) to chronic pulmonary aspergillosis (CPA), allergic bronchopulmonary aspergillosis, fungal ball of the lung (aspergilloma), fungal ball of the sinus, otomycoses, keratitis, cutaneous and wound infections, fungal allergic diseases, asthma exacerbation and severe asthma with fungal sensitization [4,5,6,7]. Although *Aspergillus fumigatus* is the primary etiological agent for human aspergillosis at most geographical locations, other *Aspergillus* species, viz., *A. flavus*, *A. terreus*, and *A. niger*, can also cause serious infections in susceptible patients [8,9].

Kuwait is a small dry and arid country in the Arabian Peninsula where dust storms are frequent, particulate matter concentration in the outdoor air and severe exacerbation rates of asthma are high, and a higher risk of all-cause mortality has also been recorded during the days of dust storms [10,11,12]. We have previously shown that spores of various filamentous fungi are present in outdoor and indoor hospital environments in Kuwait [13]. *Aspergillus* spp. were the second-most-common filamentous fungi, accounting for 22% of all cultured isolates in Kuwait [13]. Furthermore, black aspergilli (members of the *Aspergillus* section *Nigri* complex) comprised 65% of all aspergilli in Kuwait [13]. The *Aspergillus* section *Nigri* strains have previously been reported as the most common etiologic agents of otomycoses, and they are also implicated in human aspergillosis [14,15,16]. Based on phenotypic and phylogenetic characteristics, *Aspergillus* section *Nigri* complex members belong to at least 27 species, which include *A. niger sensu stricto* (*A. niger*), *A. tubingensis*, *A. awamori*, *A. welwitschiae*, and several other cryptic species [8,14,17,18]. The *Aspergillus* section *Nigri* complex members also differ in their pathogenicity and susceptibility to antifungal drugs [8,14,17,18]. Thus, accurate identification of *Aspergillus* section *Nigri* complex members and their susceptibility to antifungal drugs helps in proper management of patients with appropriate antifungal drugs [8,18,19]. However, there is no information on the species spectrum of *Aspergillus* section *Nigri* complex members isolated from clinical specimens in Kuwait or from other countries in the Arabian Peninsula. This study describes the relative prevalence of different *Aspergillus* section *Nigri* complex species among clinical isolates and their susceptibility to antifungal drugs in Kuwait.

## 2. Materials and Methods

### 2.1. Clinical and Environmental Isolates

This retrospective study is based on a total of 34 clinical strains of *Aspergillus* section *Nigri* (CASN1 to CASN34) isolated from various human specimens (respiratory, n = 20; ear discharge, n = 7; sinus/nasal tissue, n = 6; wound pus, n = 1) and two environmental isolates (EASN1 and EASN2) randomly selected from the stock culture collection maintained within the Mycology Reference Laboratory, Department of Microbiology, Faculty of Medicine, Kuwait University, Safat, Kuwait. The specimens yielding clinical isolates were obtained from 2014 to 2017 from patients visiting or hospitalized in nine different hospitals (coded as A to I) across Kuwait during routine patient care for fungal infections. The results described in this paper are fully anonymized. The study was approved by the Ethical Committee of the Health Sciences Center, Kuwait University (Approval no. VDR/EC-386 dated 31 May 2023). The two environmental isolates were cultured from outdoor air, as described previously [13].

### 2.2. Phenotypic and Molecular Identification

The isolates were initially identified as belonging to *Aspergillus* section *Nigri* complex based on macroscopic colony characteristics (such as colony shape, diameter, texture, surface color and its alteration, etc.) and microscopic morphological characteristics (such as size, shape, color and surface ornamentation of conidia, appearance of conidial heads, seriation, vesicle shape, size, etc.), as described previously [20,21]. Since the internal transcribed spacer (ITS) region of rDNA is not sufficiently polymorphic in the genus *Aspergillus*, the variable region of the β-tubulin (*benA*) gene and calmodulin (*caM*) gene sequences are used for the taxonomy of *Aspergillus* spp. [8,22,23]. Thus, molecular identification of all (n = 36) isolates was achieved by partial sequencing of the β-tubulin (*benA*) gene. For this purpose, the isolates, stored at −80 °C, were subcultured on Sabouraud dextrose agar plates to ensure purity, and genomic DNA was extracted as described previously [24]. Briefly, the mycelial mat from each isolate was transferred into 50-mL polypropylene screw cap tubes containing 6 to 8 (4 mm diameter) glass beads; the tubes were immersed in liquid nitrogen for 10 s, vortexed vigorously for 30 s, and the DNA was extracted using phenol–chloroform–isoamyl alcohol, as described in detail previously [24]. The final DNA concentration was determined by its absorbance at 260 and 280 nm, and typically, 2 µL was used for PCR amplification of various gene targets. The β-tubulin gene fragment was amplified by PCR with BTUBF (5′-TGGTAACCAAATCGGTGCTGCTT-3′) and BTUBR (5′-GCACCCTCAGTGTAGTGACCCT-3′) primers [24]. The PCR amplification was carried out in a total volume of 50 µL containing 1x AmpliTaq PCR buffer I, 1 U AmpliTaq DNA polymerase (Applied Biosystems Inc., Foster City, CA, USA), 5 pmol each of BTUBF and BTUBR primers, 2 μL of genomic DNA, and 0.1 mM of each dNTP. PCR cycling conditions included an initial denaturation step at 95 °C for 5 min, 30 cycles of denaturation at 95 °C for 1 min, primer annealing at 55 °C for 30 s, and extension at 72 °C for 1 min, followed by a final extension at 72 °C for 10 min. A portion (10 µL) of the PCR products was run on 2% agarose gels to confirm the amplification of the expected (~560 bp) PCR fragment [24], while the amplicons in the remaining (40 µL) portion were purified by using the PCR product purification kit (Qiagen, Hilden, Germany) according to kit instructions. Both strands of the purified amplicons were sequenced by using internal primers (BTUBFS, 5′-TAACCAAATCGGTGCTGCTTTCTG-3′ or BTUBRS, 5′-CCTCAGTGTAGTGACCCTTGGC-3′) and the BigDye terminator (version 3.1) cycle sequencing kit (Applied Biosystems Inc.) according to kit instructions and as described previously [24]. The primers and unincorporated nucleotides were removed from the sequencing products by using the BigDye X-terminator kit, and the samples were then loaded on the ABI 3130*xl* Genetic Analyzer (Applied Biosystems Inc.) by following the manufacturer’s instructions. The sequence data were assembled, ensuring that the forward- and reverse-primer-generated sequences were 100% complementary, and then GenBank Basic Local Alignment Search Tool (BLAST) searches (https://blast.ncbi.nlm.nih.gov/Blast.cgi?, accessed on 16 February 2026) were performed for species-specific identification. A sequence identity >99% was used to define species within the *Aspergillus* section *Nigri* complex.

The variable region of the calmodulin (caM) gene was also amplified from 20 of the above 36 isolates (due to lack of sufficient funds) by using Cmd5 (5′-GTCTCCGAGTACAAGGAGGC-3′) and Cmd6 (5′-TCGCCGATRGAGGTCATRACGTG-3′) primers and the purified amplicons were sequenced with internal primers (CMDFS, 5′-TCCGAGTACAAGGAGGCCTTC-3′ or CMDRS, 5′-GATAGAGGTCATRACGTGRCGCA-3′) and the data were assembled as described above for the β-tubulin gene fragment.

### 2.3. Phylogenetic Analysis of Aspergillus Section Nigri Isolates

A phylogenetic analysis was performed using the Maximum Likelihood method with β-tubulin sequences from 36 clinical and environmental isolates of *Aspergillus* section *Nigri*. The dendrogram was generated in MEGA software (version 11; https://www.megasoftware.net/ accessed on 31 May 2026) using the Maximum Likelihood method and the Tamura–Nei evolutionary model, with the pairwise deletion of gaps option, as described previously [25]. The robustness of the tree topology was evaluated by bootstrap analysis with 1000 replicates [25]. DNA sequence data for reference strains of *A. niger* CBS55465 (GenBank Accession no. AY585536) and CBS117.80 (GenBank Accession no. OP081891), *A. tubingensis* CBS122719 (GenBank Accession no. EU600389), and *A. luchuensis* CBS56465 (GenBank Accession no. AY585533) were also included. *Aspergillus flavus* CBS100927 (GenBank Accession no. EF203132) was used as an outgroup.

The calmodulin gene sequence data for the subset of 20 isolates were also used to construct a phylogenetic tree using the Maximum Likelihood method, as described above for the β-tubulin gene. DNA sequence data for reference strains of *A. niger* CBS121.55 (GenBank Accession no. KM593228), *A. tubingensis* CBS119382 (GenBank Accession no. PV012874), and *A. luchuensis* KACC45131 (GenBank Accession no. JX500074) were also included. *Aspergillus flavus* CBS100927 (GenBank Accession no. EF202063) was used as an outgroup.

### 2.4. Antifungal Drug Susceptibility Testing

Antifungal susceptibility testing of clinical and environmental *Aspergillus* section *Nigri* complex isolates to five antifungal drugs (amphotericin B, itraconazole, voriconazole, posaconazole, and caspofungin) was carried out using Etest (AB Biodisk, bioMérieux, Marcy-l’Étoile, France) according to the manufacturer’s instructions and as described previously [26]. Briefly, each isolate was freshly sub-cultured on potato dextrose agar to obtain good sporulation, the spores were suspended in 0.9% saline and 0.2% Tween 80 and the conidial suspension was evenly spread on Roswell Park Memorial Institute (RPMI) 1640 medium (pH 7.0) agar plates supplemented with 2% glucose and the minimum inhibitory concentration (MIC) values were read after 24 h at 35 °C. Reference strains *C. parapsilosis* (ATCC22019), *C. krusei* (ATCC6258), and *A. fumigatus* (CBS113.26) were also simultaneously tested for quality control. The proposed European Committee on Antimicrobial Susceptibility Testing (EUCAST) epidemiological cutoff values of 0.25, 4.0, 2.0, 0.5, and 0.25 µg/mL were used for the interpretation of susceptibility testing data for amphotericin B, itraconazole, voriconazole, posaconazole, and caspofungin, respectively [18,27,28].

## 3. Results

A total of 34 strains of black aspergilli (CASN1 to CASN34) isolated from various human clinical specimens and two environmental (outdoor air) (EASN1 and EASN2) isolates were used. The clinical isolates were cultured from sputum (n = 11), ear discharge (n = 7), sinus/nasal tissue biopsy (n = 6), endotracheal aspirate (n = 5), bronchoalveolar lavage (n = 3), tracheal secretion (n = 1), and wound pus (n = 1) (Table 1). During phenotypic identification, our isolates produced similar colony characteristics on Sabouraud dextrose agar plates, initially appearing as white mycelium before turning dark brown to black. Microscopic features included spherical vesicles and biseriate phialides, which produced dark-brown to black conidia with a spiny appearance. These phenotypic features were not adequate for species-specific identification of our isolates. Based on β-tubulin gene sequence comparisons, three species were detected among 36 *Aspergillus* section *Nigri* strains from Kuwait: 27 isolates identified as *A. niger* (including all 7 isolates from ear discharge), 8 isolates identified as *A. tubingensis*, and 1 isolate recovered from a nasal tissue biopsy identified as *A. luchuensis* (Table 1).

**Figure 1:**
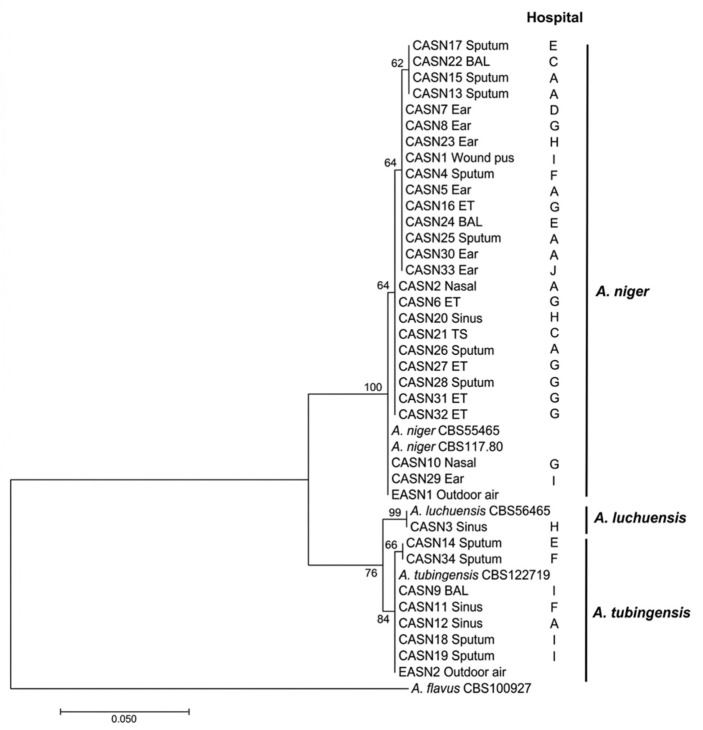
Phylogenetic analysis of 34 clinical (CASN) and 2 environmental (EASN) *Aspergillus* section *Nigri* isolates from Kuwait. The dendrogram is based on partial β-tubulin gene sequences from Kuwait in combination with reference *Aspergillus* spp. strains and Maximum Likelihood clustering. Species-specific identification of *A. niger*, *A. luchuensis*, and *A. tubingensis* is indicated on the right. Reference *A. flavus* strain CBS100927 was used as an outgroup. Evolutionary distances are indicated by the scale bar (0.050 substitutions per site). Bootstrap support values are shown at the internal nodes. The source of isolation and the patient’s hospital are shown for each clinical isolate. BAL, bronchoalveolar lavage; Ear, ear discharge; ET, endotracheal aspirate; Nasal, nasal tissue biopsy; Sinus, sinus tissue biopsy; TS, tracheal secretion.

The β-tubulin gene sequences were also used to determine genetic heterogeneity among clinical *Aspergillus* section *Nigri* isolates from Kuwait by constructing a phylogenetic tree using the Maximum Likelihood method. The dendrogram is shown in Figure 1. The phylogenetic analysis grouped our isolates with the reference sequences of *A. niger*, *A. luchuensis*, and *A. tubingensis*. The phylogenetic tree also showed genetic heterogeneity among *A. niger* and *A. tubingensis* isolates from Kuwait. Thus, four sequence patterns were identified among 26 clinical *A. niger* isolates, while two sequence patterns were identified among 7 clinical *A. tubingensis* isolates. However, there was no specific relationship between a specific sequence pattern and the source of isolation or the hospital from which the clinical specimens were collected. The calmodulin gene fragment was also sequenced but only from 20 of 36 isolates due to a lack of funds and yielded concordant species-specific identification results with those obtained with β-tubulin gene sequences for all 20 isolates. The dendrogram (Supplementary Figure S1) based on calmodulin gene sequences also grouped our isolates with the reference sequences of *A. niger*, *A. luchuensis*, and *A. tubingensis*.

The antifungal susceptibility testing data for 27 *A. niger*, 8 *A. tubingensis*, and 1 *A. luchuensis* isolates are presented in Table 2. Based on the epidemiological cutoff values, all 27 *A. niger*, 8 *A. tubingensis*, and the single *A. luchuensis* isolate from Kuwait appeared susceptible to all five (amphotericin B, itraconazole, voriconazole, posaconazole, and caspofungin) antifungal drugs tested. Although 4 of 8 (50%) *A. tubingensis* isolates exhibited higher (>1.0) MIC and higher geometric mean (1.225 µg/mL) against itraconazole compared to only 5 of 27 (18.5%) *A. niger* isolates with a geometric mean of 0.803 µg/mL, they were all below the epidemiological cutoff value of 4 µg/mL (Table 2).

## 4. Discussion

*Aspergillus* section *Nigri* complex members differ in their pathogenicity and susceptibility to antifungal drugs [8,14,17,18]. Accurate identification and antifungal susceptibility testing of clinical *Aspergillus* spp. isolates is crucial in clinical settings for proper patient management [18,19]. However, phenotypic methods alone are not sufficient for accurate, species-specific identification of *Aspergillus* section *Nigri* complex members [20,21]. Previous studies have shown that the sequence data for the variable region of β-tubulin (*benA*) and calmodulin (*caM*) genes are more useful for molecular taxonomy of *Aspergillus* spp. since the commonly used internal transcribed spacer (ITS) region of rDNA is not sufficiently polymorphic in the genus *Aspergillus* [8,22,23]. The β-tubulin (*benA*) gene sequence data for black aspergilli (n = 34) from clinical specimens in Kuwait showed that *A. niger* (n = 26) was the dominant species, followed by *A. tubingensis* (n = 7), while one isolate was identified as *A. luchuensis* (also known as *A. acidus*) [29]. Unlike other studies (detailed below), all seven ear discharge samples yielded *A. niger*. Other common species of the *Aspergillus* section *Nigri* complex, such as *A. welwitschiae*, were not detected in Kuwait. There is considerable diversity in the species spectrum of clinical *Aspergillus* section *Nigri* complex strains from different geographical locations around the world. Three recent studies have reported the species spectrum of *Aspergillus* section *Nigri* complex in clinical specimens from nearby Iran with considerable species diversity [16,30,31]. Kamali Sarwestani et al. [16] detected only *A. tubingensis* (n = 32) and *A. niger* (n = 11) among 43 isolates collected from otomycosis cases from Tehran, Iran. On the contrary, *A. welwitschiae* was the dominant species (n = 52) followed by *A. tubingensis* (n = 31) while only 2 *A. niger* were detected among 86 *Aspergillus* section *Nigri* complex strains collected from otomycoses cases in Ahvaz, Iran [30]. *A. welwitschiae* was also the dominant species (n = 52) followed by *A. tubingensis* (n = 41) and *A. niger* (n = 20) in a more recent study involving 118 *Aspergillus* section *Nigri* complex strains collected from ear discharge (n = 98), bronchoalveolar lavage (BAL) (n = 18) and sputum (n = 2) samples in seven provinces (including Ahvaz and three other provinces nearby Kuwait) in Iran [31]. Interestingly, *A. luchuensis* was also detected in two bronchoalveolar lavage (BAL) samples in this study [31].

Studies from other geographical locations have also shown considerable diversity. In a study from Manchester, UK, *A. awamori* was the dominant species (n = 25), followed by *A. tubingensis* (n = 8), *A. niger* (n = 6), and *A. luchuensis* (n = 3), among 45 clinical *Aspergillus* section *Nigri* complex strains [32]. Similarly, *A. awamori* was the dominant species (n = 7), closely followed by *A. tubingensis* (n = 6) and *A. niger* (n = 3), among 16 clinical *Aspergillus* section *Nigri* complex strains in Italy [33], while *A. niger* was the dominant species (n = 128), followed by *A. tubingensis* (n = 79), among 212 clinical *Aspergillus* section *Nigri* complex strains in France [8]. Gautier et al. [8,34] have also shown that *A. tubingensis* is a significant contributor to human lung disease in France but can also cause otomycoses. In Japan, *A. welwitschiae* was the dominant species (n = 22), followed by *A. tubingensis* (n = 17) and *A. niger* (n = 4), among 43 *Aspergillus* section *Nigri* complex strains [28]. These differences could be attributed to the relative prevalence of different black aspergilli at various geographical locations due to different environmental factors, such as humid and temperate versus dry and arid conditions.

Consistent with the worldwide trends [18,32,35], the geometric mean MIC values were slightly higher for *A. tubingensis* than for *A. niger* isolates for itraconazole and voriconazole. However, all 27 *A. niger*, 8 *A. tubingensis*, and 1 *A. luchuensis* isolates appeared susceptible to all three triazoles and amphotericin B. Although few *A. tubingensis* isolates yielded higher MIC values, all 31 *A. tubingensis* and 2 *A. niger* isolates in the study from Ahvaz, Iran, were also susceptible to itraconazole and voriconazole, while 1 *A. tubingensis* isolate was non-wild-type for amphotericin B [30]. Other studies from Europe or Asia on clinical *Aspergillus* section *Nigri* complex members, particularly *A. tubingensis* strains, have reported variable reduced susceptibility/resistance to triazoles and/or amphotericin B in some isolates. For instance, all 5 invasive *A. tubingensis* isolates from Belgium were resistant (MIC range 4–8 µg/mL) to itraconazole [14]. On the contrary, only 1 of 6 *A. tubingensis* isolate was resistant, while all 7 *A. awamori* and 3 *A. niger* isolates from Italy were susceptible to itraconazole [33]. The study from China has also reported itraconazole resistance in only 2 of 55 (3.6%) *Aspergillus* section *Nigri* complex strains [36]. However, the resistance to itraconazole was highly variable among clinical *Aspergillus* section *Nigri* complex members, varying from 100% in *A. luchuensis* to 90% in *A. tubingensis* to 36% in *A. awamori* and 33% in *A. niger* isolates from Manchester, UK [32]. Similarly, it was also highly variable among clinical *Aspergillus* section *Nigri* complex members, varying from 80% in *A. tubingensis* to 7% in *A. niger* and 4% in *A. welwitschiae* isolates from Chiba, Japan [37]. Similar trends were also noted in a more recent study from Japan, as 9 of 17 *A. tubingensis* but only 1 of 22 *A. welwitschiae* isolates exhibited reduced susceptibility (MICs > 2 µg/mL) to itraconazole [28]. The resistance mechanisms to azoles in *Aspergillus* section *Nigri* complex members are not completely understood [18,32,37,38]. Reduced susceptibility of some species (e.g., *A. tubingensis*) to azoles could be attributed to specific amino acid polymorphisms in *Cyp51A* gene sequences, while geographical variations in the prevalence of resistance rates could be due to variations in the use of azoles as prophylactic or empirical treatment agents [37,38,39,40].

Our study has a few limitations. The antifungal susceptibility testing was carried out by Etest and not by the reference broth microdilution method. The details, including specific clinical diagnosis, treatment given, and outcome of the patients yielding *Aspergillus* section *Nigri* complex strains, were not available. The lack of detection of some *Aspergillus* section *Nigri* complex species, such as *A. awamori* and *A. welwitschiae*, in our samples could be attributed to the relatively small number (n = 34) of clinical isolates analyzed in the study.

## 5. Conclusions

Our study, based on β-tubulin gene sequences, identified only three species: *A. niger* (n = 26), *A. tubingensis* (n = 7), and *A. luchuensis* (n = 1) among 34 clinical *Aspergillus* section *Nigri* isolates in Kuwait. Although β-tubulin gene sequences also identified four and two different sequence patterns among *A. niger* and *A. tubingensis* isolates, respectively, no specific association of a given sequence pattern with clinical source of isolation or the patient’s hospital was apparent. All isolates appeared susceptible to all five antifungal drugs tested. Our study shows that *A. niger* predominates among phenotypically identified clinical isolates of the *Aspergillus* section *Nigri* complex and is the main agent in otomycosis cases in Kuwait. The detection of only three species within the *Aspergillus* section *Nigri* complex in Kuwait could be due to the limited number (n = 34) of clinical isolates analyzed in this study.

## Figures and Tables

**Table 1:** Species distribution of *Aspergillus* section *Nigri* complex isolated from various clinical and environmental air samples.

Specimen	Total No. of	No. of Isolates Identified as		
Type	Isolates Tested	*A. niger*	*A. tubingensis*	*A. luchuensis*
Clinical				
Sputum	11	7	4	
Ear discharge	7	7		
Sinus/nasal tissue biopsy	6	3	2	1
Endotracheal aspirate	5	5		
Bronchoalveolar lavage	3	2	1	
Wound pus	1	1		
Tracheal secretion	1	1		
Environmental				
Outdoor air	2	1	1	
**Total**	**36**	**27**	**8**	**1**

**Table 2:** In vitro susceptibility of *Aspergillus* section *Nigri* complex species to five antifungal drugs.

Antifungal	*Aspergillus*	No. of Isolates with Minimum Inhibitory Concentration (MIC) (µg/mL) of															Geometric
Drug	Species	≤0.01	0.02	0.03	0.05	0.06	0.09	0.13	0.19	0.25	0.38	0.5	0.75	1	1.5	2	Mean
Amphotericin B	*A. niger* (n = 27)	1			2	3	11	6	3			1					0.095
	*A. tubingensis* (n = 8)		1		1	2	2	1	1								0.072
	*A. luchuensis* (n = 1)					1											
Itraconazole	*A. niger* (n = 27)										2	6	5	9	4	1	0.803
	*A. tubingensis* (n = 8)												2	2	2	2	1.225
	*A. luchuensis* (n = 1)									1							
Voriconazole	*A. niger* (n = 27)		1	1	2	3	5	10	2	3							0.101
	*A. tubingensis* (n = 8)					1	1	4	1		1						0.135
	*A. luchuensis* (n = 1)						1										
Posaconazole	*A. niger* (n = 27)	1	2	1	5	4	8	4	2								0.067
	*A. tubingensis* (n = 8)		1	1		5		1									0.053
	*A. luchuensis* (n = 1)	1															
Caspofungin	*A. niger* (n = 27)	23	2		1		1										0.012
	*A. tubingensis* (n = 8)	6	2														0.012
	*A. luchuensis* (n = 1)	1															

## Data Availability

The nucleotide sequence data reported in this study have been deposited to GenBank database (accession no. PZ098559–PZ098585, PZ098703–PZ098710, PZ098719, and PZ131175–PZ131194.

## Supplementary Materials

The following supporting information can be downloaded at: https://www.mdpi.com/article/10.3390/jof12060430/s1, Figure S1. Phylogenetic analysis of 20 clinical *Aspergillus* section *Nigri* isolates from Kuwait based on partial calmodulin gene sequences in combination with reference *Aspergillus* spp. strains and Maximum Likelihood clustering.

## References

[1] de S Araújo G.R., Souza W., Frases S. (2017). The hidden pathogenic potential of environmental fungi. Future Microbiol..

[2] Denham S.T., Wambaugh M.A., Brown J.C.S. (2019). How environmental fungi cause a range of clinical outcomes in susceptible hosts. J. Mol. Biol..

[3] Apostolopoulou A., Kampouri E., Little J.S. (2026). The changing epidemiology of invasive mould diseases in immunosuppressed patients: What, why, how?. Clin. Microbiol. Infect..

[4] Walsh T.J., Anaissie E.J., Denning D.W., Herbrecht R., Kontoyiannis D.P., Marr K.A., Morrison V.A., Segal B.H., Steinbach W.J., Stevens D.A. (2008). Treatment of aspergillosis: Clinical practice guidelines of the Infectious Diseases Society of America. Clin. Infect. Dis..

[5] Kosmidis C., Denning D.W. (2015). The clinical spectrum of pulmonary aspergillosis. Thorax.

[6] Evans T.J., Lawal A., Kosmidis C., Denning D.W. (2024). Chronic pulmonary aspergillosis: Clinical presentation and management. Semin. Respir. Crit. Care Med..

[7] Lieu A., Mah J.K., Vinh D.C., Qureshi S.T. (2026). Pulmonary fungal infections in the immunocompetent host. Chest.

[8] Gautier M., Normand A.-C., Ranque S. (2016). Previously unknown species of *Aspergillus*. Clin. Microbiol. Infect..

[9] Rafique A., Sharmin S., Raj A., Mohiuddin A.L., Mahmud M.I.A., Bin Md Omer H. (2026). Comparative overview of *Aspergillus fumigatus*, *A. flavus*, and *A. niger*: Pathogenicity, resistance, and public health significance. J. Infect. Public Health.

[10] Achilleos S., Al-Ozairi E., Alahmad B., Garshick E., Neophytou A.M., Bouhamra W., Yassin M.F., Koutrakis P. (2019). Acute effects of air pollution on mortality: A 17-year analysis in Kuwait. Environ. Int..

[11] Al Salameen F., Habibi N., Uddin S., Al Mataqi K., Kumar V., Al Doaij B., Al Amad S., Al Ali E., Shirshikhar F. (2020). Spatio-temporal variations in bacterial and fungal community associated with dust aerosol in Kuwait. PLoS ONE.

[12] Lee T.Y., Price D., Yadav C.P., Roy R., Lim L.H.M., Wang E., Wechsler M.E., Jackson D.J., Busby J., Heaney L.G. (2024). International variation in severe exacerbation rates in patients with severe asthma. Chest.

[13] Asadzadeh M., Ahmad S., Hagen F., Meis J.F., Khan Z. (2025). Occurrence of pathogenic and allergenic molds in the outdoor and indoor environment of a major hospital and molecular epidemiology of *Aspergillus fumigatus* in Kuwait. J. Fungi.

[14] Vermeulen E., Maertens J., Meersseman P., Saegeman V., Dupont L., Lagrou K. (2014). Invasive *Aspergillus niger* complex infections in a Belgian tertiary care hospital. Clin. Microbiol. Infect..

[15] Jia X., Liang Q., Chi F., Cao W. (2012). Otomycoses in Shanghai: Etiology, clinical features and therapy. Mycoses.

[16] Sarwestani Z.K., Hashemi S.J., Rezaie S., Shoar M.G., Mahmoudi S., Elahi M., Bahardoost M., Tajdini A., Abutalebian S., Ghazvini R.D. (2018). Species identification and in vitro antifungal susceptibility testing of *Aspergillus* section *Nigri* strains isolated from otomycoses patients. J. Mycol. Med..

[17] Vesth T.C., Nybo J.L., Theobald S., Frisvad J.C., Larsen T.O., Nielsen K.F., Hoof J.B., Brandl J., Salamov A., Riley R. (2018). Investigation of inter- and intraspecies variation through genome sequencing of *Aspergillus* section *Nigri*. Nat. Genet..

[18] Djenontin E., Lavergne R.A., Morio F., Dannaoui E. (2025). Antifungal resistance in non-*fumigatus Aspergillus* species. Mycoses.

[19] Takeda K., Suzuki J., Sasaki V., Watanabe A., Kamei K. (2023). Importance of accurate identification and antifungal susceptibility testing of *Aspergillus* species in clinical settings. Med. Mycol. J..

[20] Hageskal G., Knutsen A.K., Gaustad P., de Hoog G.S., Skaar I. (2006). Diversity and significance of mold species in Norwegian drinking water. Appl. Environ. Microbiol..

[21] Samson R.A., Visagie C.M., Houbraken J., Hong S.B., Hubka V., Klaassen C.H., Perrone G., Seifert K.A., Susca A., Tanney J.B. (2014). Phylogeny, identification and nomenclature of the genus *Aspergillus*. Stud. Mycol..

[22] Samson R.A., Noonim P., Meijer M., Houbraken J., Frisvad J.C., Varga J. (2007). Diagnostic tools to identify black aspergilli. Stud. Mycol..

[23] Varga J., Frisvad J.C., Kocsubé S., Brankovics B., Tóth B., Szigeti G., Samson R.A. (2011). New and revisited species in *Aspergillus* section *Nigri*. Stud. Mycol..

[24] Khan Z., Ahmad S., Al-Ghimlas F., Al-Mutairi S., Joseph L., Chandy R., Sutton D.A., Guarro J. (2012). *Purpureocillium lilacinum* as a cause of cavitary pulmonary disease: A new clinical presentation and observations on atypical morphologic characteristics of the isolate. J. Clin. Microbiol..

[25] Al-Otaibi H., Asadzadeh M., Ahmad S., Al-Sweih N., Joseph L. (2021). *Papiliotrema laurentii* fungemia in a premature, very low-birth-weight neonate in Kuwait successfully treated with liposomal amphotericin B. J. Mycol. Med..

[26] Al-Wathiqi F., Ahmad S., Khan Z. (2013). Molecular characterization and antifungal susceptibility profile of *Aspergillus flavus* isolates recovered from clinical specimens in Kuwait. BMC Infect. Dis..

[27] Guinea J. (2020). Updated EUCAST clinical breakpoints against *Aspergillus*, implications for the clinical microbiology laboratory. J. Fungi.

[28] Espinel-Ingroff A., Sasso M., Turnidge J., Arendrup M., Botterel F., Bourgeois N., Bouteille B., Canton E., Cassaing S., Dannaoui E. (2021). Etest ECVs/ECOFFs for detection of resistance in prevalent and three nonprevalent *Candida* spp. to triazoles and amphotericin B and *Aspergillus* spp. to caspofungin: Further assessment of modal variability. Antimicrob. Agents Chemother..

[29] Hong S.B., Lee M., Kim D.H., Varga J., Frisvad J.C., Perrone G., Gomi K., Yamada O., Machida M., Houbraken J. (2013). *Aspergillus luchuensis*, an industrially important black *Aspergillus* in East Asia. PLoS ONE.

[30] Halvaeezadeh M., Jalaee G.A., Fatahinia M., Mahmoudabadi A.Z. (2023). *Aspergillus welwitschiae*: An otomycosis predominant agent, new epidemiological and antifungal susceptibility data from Iran. Microb. Pathog..

[31] Hamzehee S., Gharaghani M., Borsi S.H., Jafarian H., Jalaee G.A., Kardooni M., Zarei-Mahmoudabadi A. (2025). The frequency distribution of *Aspergillus* section *Nigri* from clinical and environmental samples in Iran. BMC Microbiol..

[32] Howard S.J., Harrison E., Bowyer P., Varga J., Denning D.W. (2011). Cryptic species and azole resistance in the *Aspergillus niger* complex. Antimicrob. Agents Chemother..

[33] Iatta R., Nuccio F., Immidiato D., Mosca A., De Carlo C., Miragliotta G., Parisi A., Crescenzo G., Otranto D., Cafarchia C. (2016). Species distribution and in vitro susceptibility of *Aspergillus* section *Nigri* isolates from clinical and environmental settings. J. Clin. Microbiol..

[34] Gautier M., Normand A.-C., L’Ollivier C., Cassagne C., Renaud-Gaubert M., Dubus J.-C., Bregeon F., Hendrickx M., Gomez C., Ranque S. (2016). *Aspergillus tubingensis*: A major filamentous fungus found in the airways of patients with lung disease. Med. Mycol..

[35] Hendrickx M., Beguin H., Detandt M. (2012). Genetic reidentification and antifungal susceptibility testing of *Aspergillus* section *Nigri* strains of the BCCM/IHEM collection. Mycoses.

[36] Peng D., Li A., Kong M., Mao C., Sun Y., Shen M. (2024). Pathogenic *Aspergillus* strains identification and antifungal susceptibility analysis of 452 cases with otomycosis in Jingzhou, China. Mycopathologia.

[37] Hashimoto A., Hagiwara D., Watanabe A., Yahiro M., Yikelamu A., Yaguchi T., Kamei K. (2017). Drug sensitivity and resistance mechanism in *Aspergillus* section *Nigri* strains from Japan. Antimicrob. Agents Chemother..

[38] Sen P., Vijay M., Kamboj H., Gupta L., Shankar J., Vijayaraghavan P. (2024). *cyp51A* mutations, protein modeling and efflux pump gene expression reveals multifactorial complexity towards understanding *Aspergillus* section *Nigri* azole resistance mechanism. Sci. Rep..

[39] Jannik Stemler J., Többen C., Lass-Flörl C., Steinmann J., Ackermann K., Rath P.-M., Simon M., Andreas Cornely O., Koehler P. (2023). Diagnosis and treatment of invasive aspergillosis caused by non-*fumigatus Aspergillus* spp.. J. Fungi.

[40] De Francesco M.A. (2023). Drug-resistant *Aspegillus* spp.: A literature review of its resistance mechanisms and its prevalence in Europe. Pathogens.

