# Supplementary material for: Prevalence of Aspergillus Section Nigri Complex Species Isolated from Clinical Specimens in Kuwait and Their Susceptibility to Antifungal Drugs
Source: J Fungi (Basel). 2026 Jun 12;12(6):430. doi: 10.3390/jof12060430 (PMC13301367; doi:10.3390/jof12060430)
Supplement: Supplementary file 1 [file jof-12-00430-s001.zip › jof-4221001-supplementary.pdf]

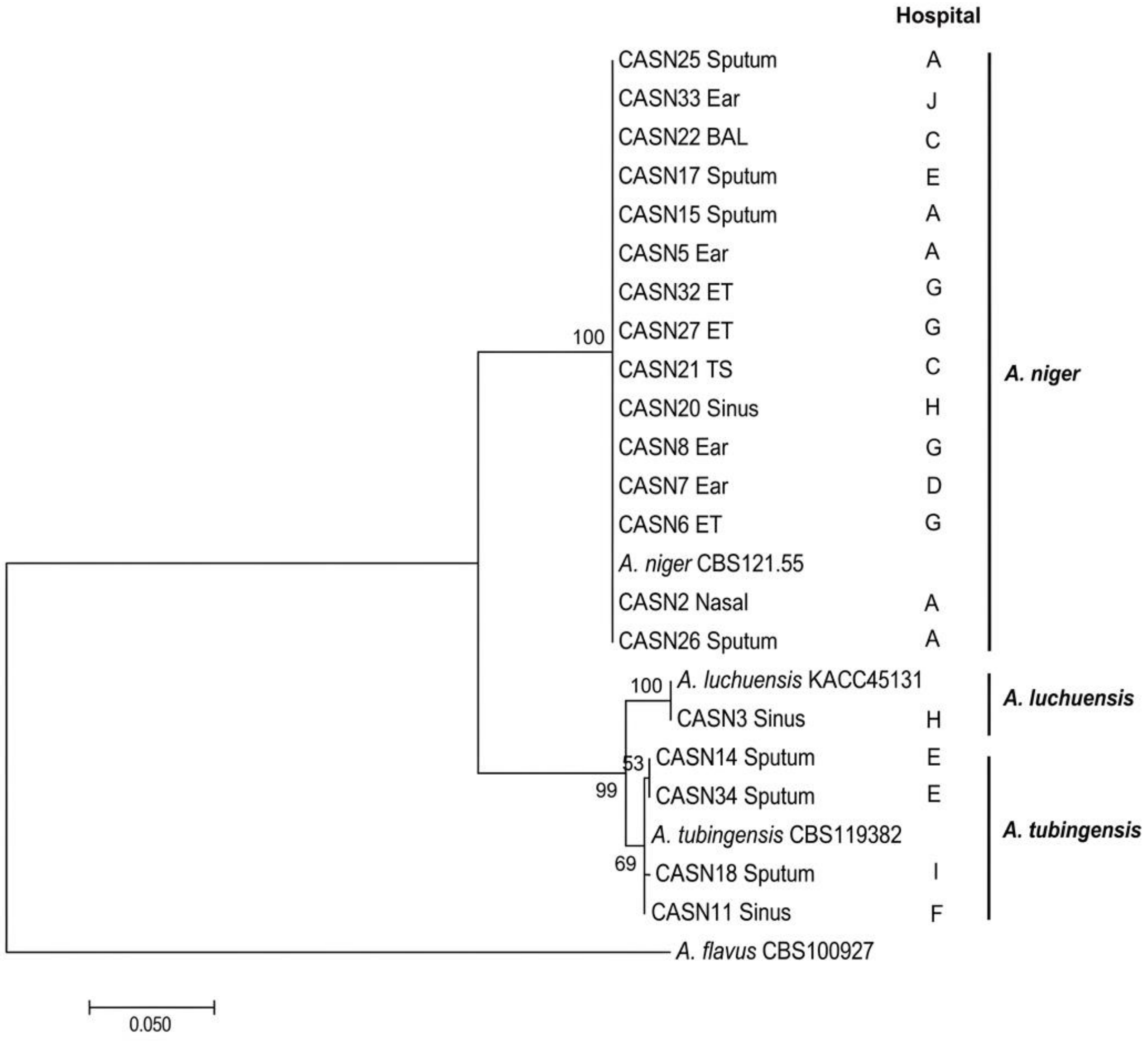

Supplementary Figure S1. Phylogenetic analysis of 20 clinical *Aspergillus* section *Nigri* isolates from Kuwait based on partial calmodulin gene sequences in combination with reference *Aspergillus* spp. strains and Maximum Likelihood clustering. Species-specific identification of *A. niger*, *A. luchuensis*, and *A. tubingensis* are indicated on the right. Reference *A. flavus* strain CBS100927 was used as an outgroup. Evolutionary distances are indicated by the scale bar. Bootstrap support values are shown at the internal nodes. The isolate (CASN) numbers refer to the same isolate as in Figure 1. The source of isolation and the patient's hospital are shown for each clinical isolate. BAL, bronchoalveolar lavage; Ear, ear discharge; ET, endotracheal aspirate; Nasal, nasal tissue biopsy; Sinus, sinus tissue biopsy; TS, tracheal secretion.
